# A Five-Gene Risk Score Model for Predicting the Prognosis of Multiple Myeloma Patients Based on Gene Expression Profiles

**DOI:** 10.3389/fgene.2021.785330

**Published:** 2021-11-30

**Authors:** Xiaotong Chen, Lintao Liu, Mengping Chen, Jing Xiang, Yike Wan, Xin Li, Jinxing Jiang, Jian Hou

**Affiliations:** Department of Hematology, Renji Hospital, Shanghai Jiao Tong University School of Medicine, Shanghai, China

**Keywords:** multiple myeloma, prognosis, risk score model, overall survival, prediction

## Abstract

Multiple myeloma is a heterogeneous plasma cell malignancy that remains incurable because of the tendency of relapse for most patients. Survival outcomes may vary widely due to patient and disease variables; therefore, it is necessary to establish a more accurate prognostic model to improve prognostic precision and guide clinical therapy. Here, we developed a risk score model based on myeloma gene expression profiles from three independent datasets: GSE6477, GSE13591, and GSE24080. In this model, highly survival-associated five genes, including *EPAS1*, *ERC2*, *PRC1*, *CSGALNACT1*, and *CCND1*, are selected by using the least absolute shrinkage and selection operator (Lasso) regression and univariate and multivariate Cox regression analyses. At last, we analyzed three validation datasets (including GSE2658, GSE136337, and MMRF datasets) to examine the prognostic efficacy of this model by dividing patients into high-risk and low-risk groups based on the median risk score. The results indicated that the survival of patients in low-risk group was greatly prolonged compared with their counterparts in the high-risk group. Therefore, the five-gene risk score model could increase the accuracy of risk stratification and provide effective prediction for the prognosis of patients and instruction for individualized clinical treatment.

## Introduction

Multiple myeloma (MM) is a heterogeneous plasma cell malignancy, which is the second most common hematological malignancy in the world (Kazandjian, 2016). There were 16,500 new cases and 10,300 deaths of MM in China in 2016; the morbidity and mortality rates increased with age, and older people were at higher risk of MM (Gerecke et al., 2016; Liu et al., 2019). The median survival in MM is approximately 6 years, with survival duration ranging from a few months to more than 10 years (Rajkumar, 2020). The International Staging System (ISS) is a widely used system for the stratification of MM patients based on easy-to-apply variables (serum beta2-microglobulin and serum albumin) (Greipp et al., 2005). In 2015, the International Myeloma Working Group (IMWG) developed the revised international staging system (R-ISS); it classifies patients into three risk groups by combining the ISS with high-risk cytogenetic abnormalities (CA) [del (17p), t (4; 14) (p16; q32), or t (14; 16) (q32; q23)] and serum lactate dehydrogenase (LDH) (Palumbo et al., 2015). For cytogenetic changes, t (4; 14), t (14; 16), t (14; 20), del (17p), and hypodiploidy have been found to be associated with high-risk diseases (Rajkumar et al., 2013; Lakshman et al., 2018).

Over the past 15 years, the survival rate of MM has improved significantly (Kumar et al., 2014). Bortezomib, lenalidomide, and dexamethasone (VRd) is the current standard of treatment for newly diagnosed MM (Rajkumar, 2020). For the treatment of recurrent MM, proteasome inhibitors, immunomodulatory substances, and classical chemotherapy agents are the main therapeutic measures (Gerecke et al., 2016). But even under these treatments, almost all patients with MM eventually relapse; the survival outcomes of MM are highly heterogeneous. Therefore, it is important to perform risk stratification for patients with MM and to find reliable prognostic biomarkers, which can better improve prognostic accuracy and guide clinical treatment (Shaughnessy et al., 2007). Comprehensive clinical information and gene expression data in public biological databases can provide opportunities to identify the prognostic signature for MM, and the biomarkers which are associated with prognostic and survival outcomes can be identified based on the gene expression of myeloma patient tissues. Studies have demonstrated that prognostic models based on the gene expression signature can predict survival outcomes in multiple independent datasets, and the discriminatory ability was also better than other combinations of traditional risk scores ( Heuck et al., 2014 ). In addition, further validation and analysis can also be performed in combination with other clinical information from more cohorts, to provide new insights for clinical application (Cai et al., 2020).

The gene expression profiles can be used to calculate differentially expressed genes (DEGs) between myeloma patients and healthy individuals, and the DEGs may associate with the prognosis of MM patients (Alizadeh et al., 2000). Some studies have already investigated the use of gene expression profiles alone or in combination with clinical factors as an improvement to estimate patient survival risk (Fernandez-Teijeiro et al., 2004; Habermann et al., 2008). The least absolute shrinkage and selection operator (Lasso) regression is a method for variable selection; it reduces the number of variables and only retains the most influential variables by using dimensionality reduction techniques. Univariate and multivariate Cox regression analyses were performed to obtain the genes correlated with prognosis in order to produce an accurate and refined model.

In this study, we integrated multiple datasets to develop and validate an effective prognostic risk model for MM patients, which was successfully validated in additional three independent datasets to demonstrate the stability and reliability of the risk model. This model contributes to risk stratification, providing important implications for the prognosis of MM and may offer the prospect of personalized therapeutic.

## Materials and Methods

### MM Dataset

**FIGURE 1:**
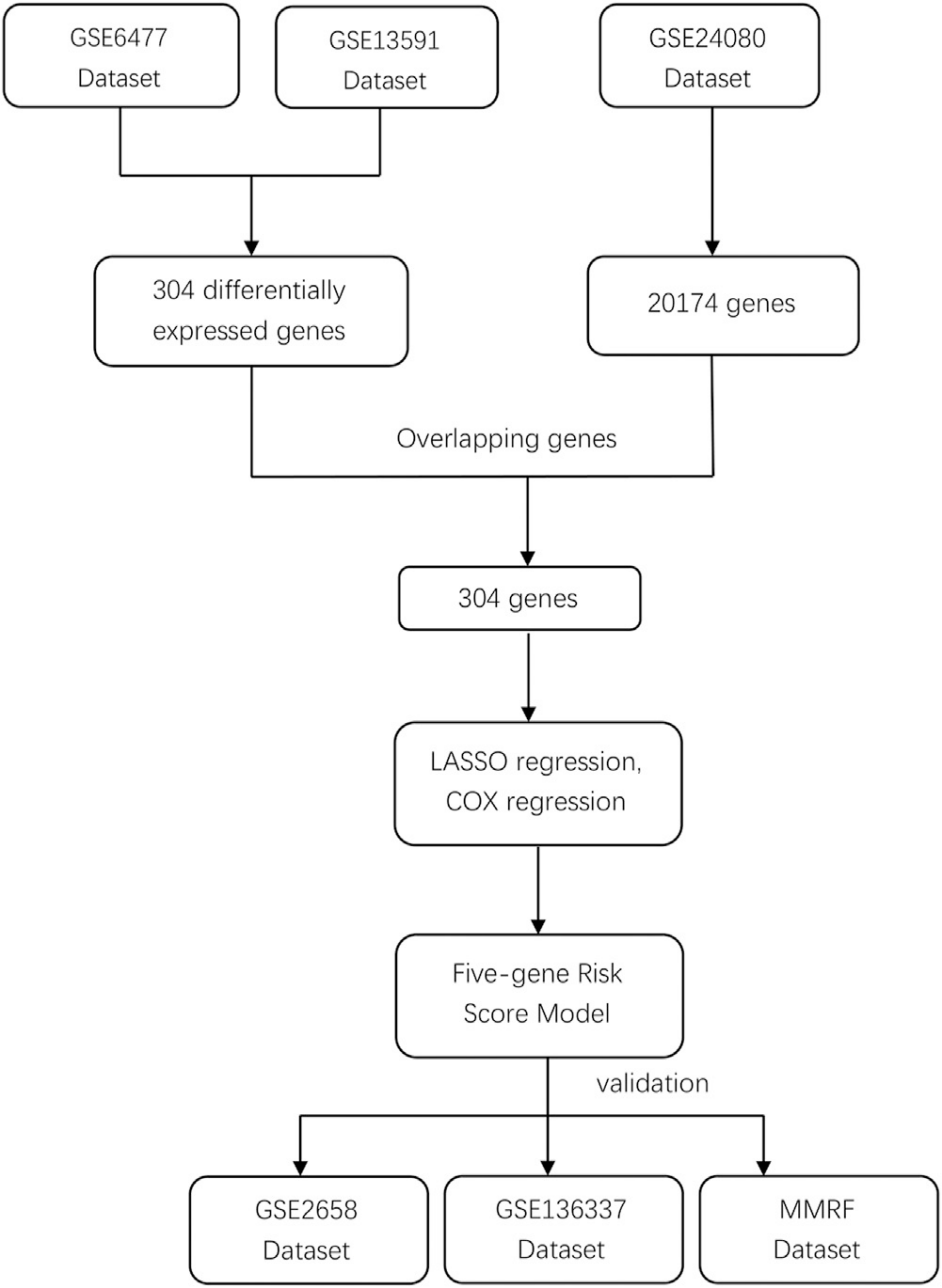
Study flowchart of prognostic model building and validation.

We systematically searched for MM datasets that were publicly available and provided prognosis information, and the datasets must have complete survival data, including survival status and OS/PFS/EFS time. For this study, GSE6477, GSE13591, GSE24080, GSE2658, and GSE136337 were downloaded from the Gene Expression Omnibus (GEO) database (https://www.ncbi.nlm.nih.gov/geo/) and normalized between different arrays. Detailed clinic-cytogenetic information including t (4; 14), t (14; 16), del (17p), ISS, and R-ISS stage and survival data were included in the datasets. The MMRF CoMMpass study is a clinical trial of newly diagnosed MM patients sponsored by the Multiple Myeloma Research Foundation; the project provides clinical information (including survival data) and the expression profile data of MM patients. Because both GSE6477 and GSE13591 contained healthy individuals and MM patients, the microarray data from these two databases were used to obtain DEGs between the two groups. GSE24080, as a training dataset, contained 559 MM patients, while the testing datasets GSE2658, GSE136337, and MMRF contained 559, 256, and 559 MM patients, respectively. The study workflow is shown in Figure 1.

### Acquisition of Prognostic Associated Genes

From GSE6477 and GSE13591 dataset, healthy individuals and MM patients were screened out, and the data were combined to create an expression matrix including 20 healthy individuals and 206 MM patients. Then, we acquired the DEGs between the two groups, genes with |log fold change| > 1 and Benjamini–Hochberg–adjusted *p* < 0.05 were considered as significant DEGs. At last, the DEGs were overlapped with 20,174 genes in the GSE24080 dataset to generate the matrix of prognostic model–associated genes.

### Lasso Regression Analysis

The GSE24080 dataset was used as the training dataset to construct the risk score model. In order to improve the prediction accuracy and interpretability of the prognostic model, Lasso regression was used to select potential prognostic genes. In this result, all genes have their own relative coefficients, and with the continuous selection and simulation of significant features, we can acquire an optimal model with the parameter lambda.min which contains the top features for constructing the risk score model. In the receiver operating characteristic (ROC) curve, the survival outcome was predicted by the patients’ risk score, and the area under curve (AUC) of the model was used to demonstrate the prediction ability of the Lasso model.

### Construction and Validation of Prognostic Risk Score Model

We used the univariate Cox regression to select genes correlated with prognosis (*p* < 0.05). Next, the Kaplan–Meier analysis was performed to screen for the genes significantly associated with survival outcome. Then, multivariate Cox regression was used to analyze these key genes (*p* < 0.05) prior to establishing the prognostic risk score model. The risk scores of all samples were calculated according to the equation: risk score = ∑coefficient value ∗ expression level.

The GSE2658, GSE136337 and MMRF datasets, were used for validation. With the median risk scores as the cut-off value to classify the high-risk and low-risk groups, we used the log-rank test to compare the difference between the two groups in both training and testing datasets.

### Statistical Analysis

The R software “sva” package was used to perform correction and acquire the integrated expression matrix by removing batch effects between different datasets. The R software “Limma” package was used to obtain DEGs, and the “Glmnet” package was used to further construct the model. Survival was compared using the Kaplan–Meier analysis with log-rank tests. Univariate and multivariate Cox regression analyses were performed for subsequent analyses. The R software “ggpubr” package was used to visualize the risk scores of patients in different survival states with the Wilcoxon tests. The R software “survival” and “survminer” packages were used to divide the patients into high-risk and low-risk groups by the median risk score. The R software vision 3.6.3 was used for statistical analyses. A two-sided *p* < 0.05 was considered statistically significant.

## Results

### Identification of Prognostic Model–Associated Genes

To develop the prognostic model for MM, GSE6477 dataset and GSE13591 dataset were used to acquire the DEGs between MM patients and healthy individuals. A total of 304 DEGs were identified with the cutoffs of |log fold change| > 1 and Benjamini–Hochberg–adjusted *p* < 0.05, among which, 90 genes were upregulated and 214 genes were downregulated, as shown in the volcano plot (Supplementary Figure S1). Next, the GSE24080 dataset was used as the training dataset and to construct the prognostic model, 20,174 genes from 559 MM patients were obtained from it. By overlapping these genes with the 304 DEGs obtained above, we acquired a matrix containing 304 genes as the prognostic model–associated genes (Supplementary Table S1).

### Construction of Lasso Regression Model

**FIGURE 2:**
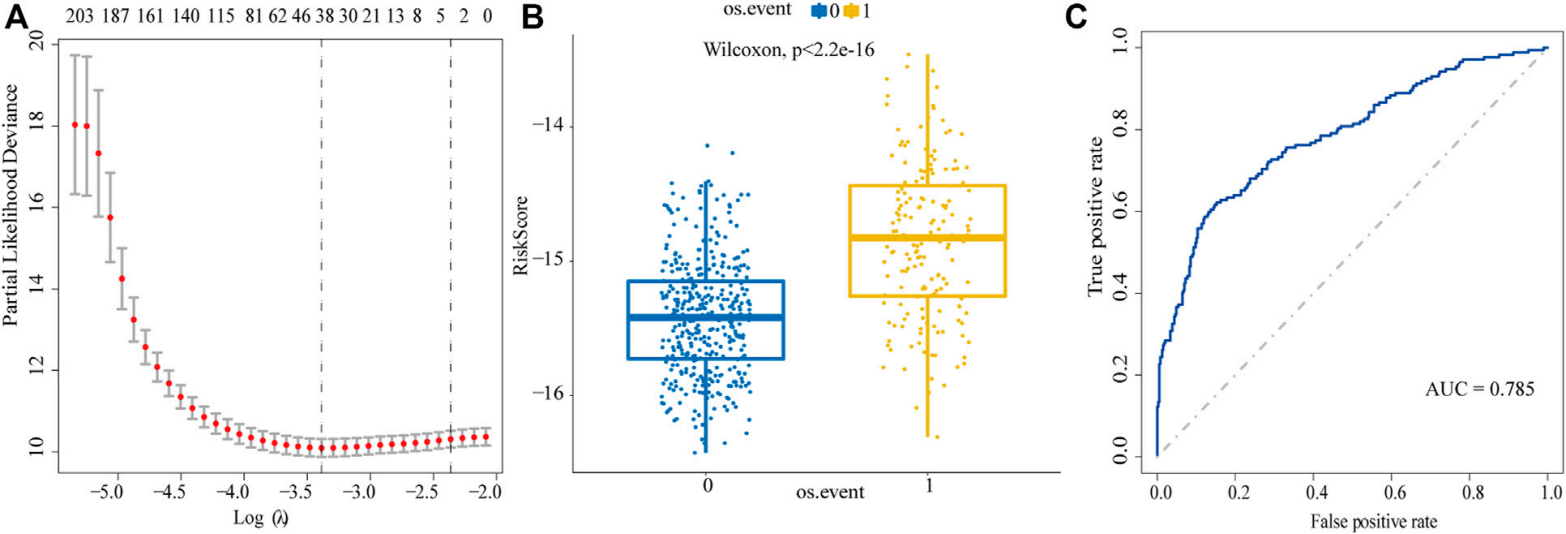
Screen prognostic genes by Lasso regression analysis. **(A)** Acquisition of the best Lambda value. The left dashed line represents lambda.min, the right dashed line represents lambda.1se. **(B)** To distinguish the survival and death events by the model based on lambda.min. 0: alive, 1: death. **(C)** ROC curve is used to evaluate the predictive performance of the model.

We used Lasso regression to select the prognostic-related genes. In this regression, the contributions of all the genes were weighted by their relative coefficients. Cross validation was used to get the best performance of the model; the left dashed line represents lambda.min, which was utilized to generate the most accurate model by minimizing the prediction error (Figure 2A). Finally, 304 genes were narrowed down to 38 potential predictor variables (Supplementary Table S2) with nonzero coefficients in the Lasso regression model. The final risk score can be acquired by multiplying the expression of each gene with its corresponding coefficient and adding them together; then, we used the median of the risk score as the cut-off value to divide the high-risk and low-risk groups. By comparing the two groups, we found the 38-gene predictive model could distinguish the survival and death events effectively (Wilcoxon test *p* < 2.2e-16, Figure 2B). In the ROC curve, the area under curve (AUC) of the predictive model is 0.785, indicating that the predictive ability of the model is favorable (Figure 2C).

### Construction of the Prognostic Model

**FIGURE 3:**
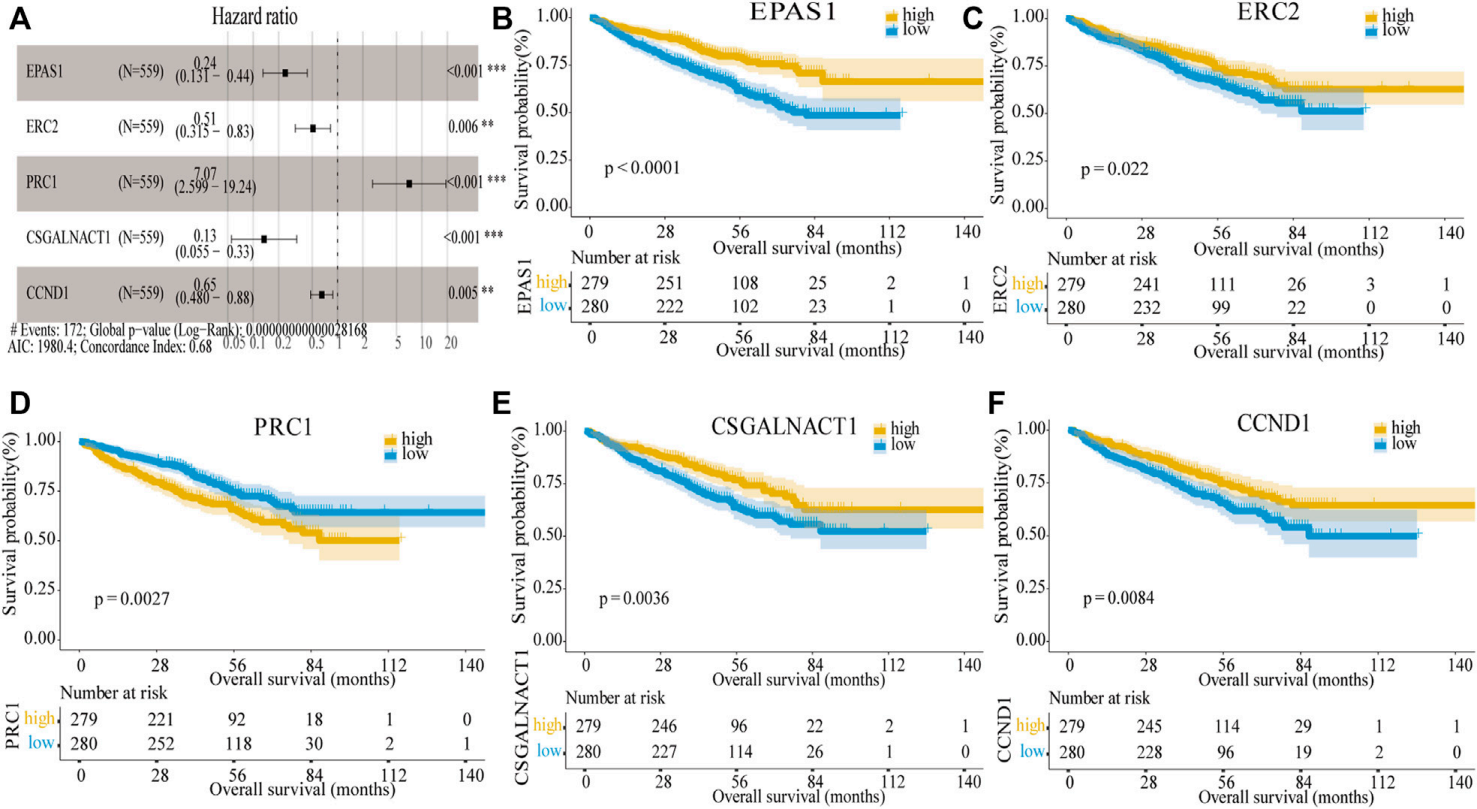
Construction of the five-gene risk score model. **(A)** Multivariate Cox regression analysis of the five genes (**p < 0.01 and ***p < 0.001). The figure also showed Hazard ratio, Global log-rank p, C-index, and AIC. **(B–F)** Kaplan–Meier survival of five prognostic genes: *EPAS1*, *ERC2*, *PRC1*, *CSGALNACT1*, and *CCND1*.

Next, the univariate Cox regression analysis and Kaplan–Meier survival analysis were used to filter the target genes, and the results showed 20 genes (Supplementary Table S3) in the GSE24080 dataset were significantly associated with prognosis. To further screen the key genes, we performed multivariate Cox regression analysis of the 20 genes, and finally we obtained a 11-gene prognostic model which was significantly associated with the prognosis in MM patients (Supplementary Table S4). We further screened the 11 genes and ordered them according to the *p* value. The data indicated that the log-rank *p* values of all genes in the model were minimal (log-rank *p* < 0.01), when containing the top four or top five genes. To obtain the optimal model, we compared the prediction results of the four-gene model and the five-gene model for the prognosis of MM. Results showed that when applying the four-gene model in the testing dataset (GSE136337), Kaplan–Meier curves of PFS showed no difference between the high-risk and low-risk groups divided by the median risk score (log-rank p = 0.092, Supplementary Figure S1B), which indicated that the four-gene model was not as valid as the five-gene model; the five-gene model can predict the prognostic outcome more effectively. Therefore, the five genes were used to build a risk score model, including *EPAS1, ERC2, PRC1, CSGALNACT1,* and *CCND1,* the multivariate Cox regression analysis showed them significantly associated with prognosis in MM patients (Figure 3A). Among them, *EPAS1, ERC2, CSGALNACT1*, and *CCND* (hazard ratio <1) were protective genes, while *PRC1* was a harmful gene (hazard ratio >1). Then MM patients were divided into the high-risk and low-risk groups by the median risk score, and the KM analysis was used to compare the overall survival (OS) difference between the two groups. As shown in Figures 3B–F, we found that the differences between each gene’s two groups were highly significant. The high expressions of *PRC1* were infaust for survival outcome in MM patients, but other genes are beneficial.

**FIGURE 4:**
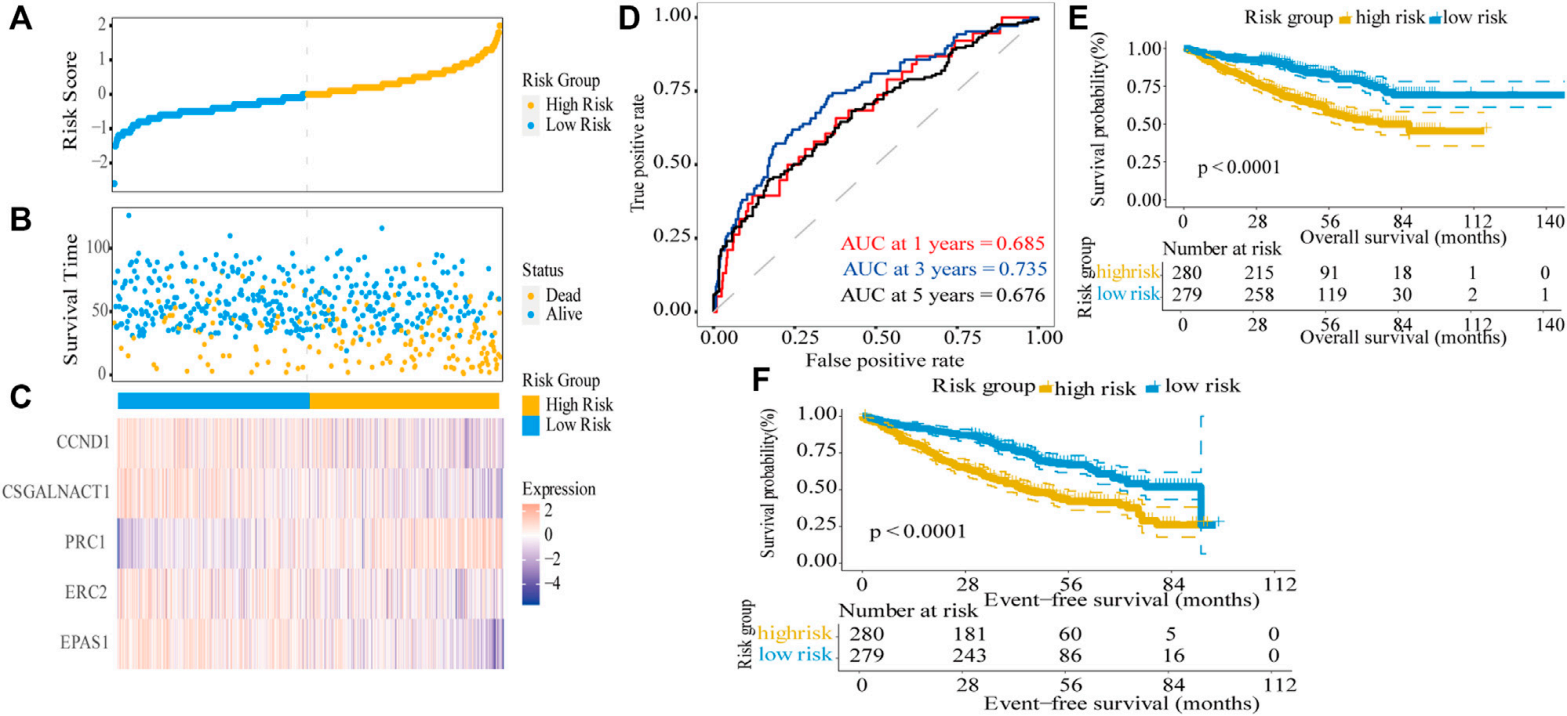
Risk score model based on five-gene signature in the training dataset. **(A)** MM patients were divided into high-risk and low-risk groups based on the median risk score. **(B)** Scatter plot of survival time and status in the high-risk and low-risk groups. **(C)** Gene expression heat map for five prognostic genes in the high-risk and low-risk groups. **(D)** Assessment of the predictive ability of the model by time-dependent ROC analysis. **(E,F)** Kaplan–Meier curves of overall survival and event-free survival for the high-risk group and low-risk group in MM patients (log-rank test, *p* < 0.0001).

Next, the dot plots were used to compare the survival of patients in the high-risk and low-risk groups and found that the survival of the low-risk group was higher than the survival of the high-risk group (Figures 4A,B). The gene expression levels in the heat map showed that four genes were decreased in the high-risk group (Figure 4C) and consistent with their hazard ratio (HR) values (HR < 1). The time-dependent ROC analysis was used to assess the predictive ability of the model, the AUC values for 1-year, 3-year, and 5-year survival were 0.685, 0.735, and 0.676, respectively (Figure 4D). In the GSE24080 dataset, to verify the predictive ability of the five-gene risk score model in the training dataset, the patients were divided into high-risk and low-risk groups by the median risk score. We found the difference between the two groups was highly significant in event-free survival (EFS) and OS (log-rank *p* < 0.0001; Figures 4E,F). The results demonstrated that the five-gene prognostic model was significantly associated with prognosis in MM patients.

### Validation of the Five-Gene Risk Score Model

**FIGURE 5:**
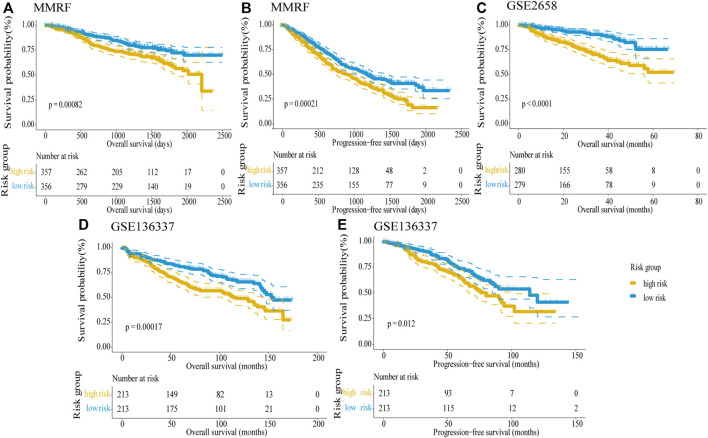
Validation of the five-gene risk score model in the testing datasets by Kaplan–Meier curves. **(A)** OS in the MMRF dataset (*p* = 0.00082). **(B)** PFS in the MMRF dataset (*p* = 0.00021). **(C)** OS in GSE2658 (*p* < 0.0001). **(D)** OS in GSE136337 (*p* = 0.00017). **(E)** PFS in GSE136337 (*p* = 0.012). MM patients were divided into high-risk and low-risk groups by the median risk score. The difference between the two groups was tested by the log-rank test.

In order to validate the predictive ability of the five-gene risk score model, we analyzed three testing datasets: the MMRF dataset, GSE2658, and GSE136337 datasets. In these testing datasets, the median risk score was taken as the cut-off value to divide high-risk and low-risk groups; then Kaplan–Meier survival curves were used to distinguish the differences between the two groups in MM patients. In the MMRF dataset, the survival information contains OS and progress-free survival (PFS), both survival information could be used to verify the five-gene risk score model. The results showed that the differences between the two groups were highly significant in OS (log-rank *p* < 0.001) as well as PFS (log-rank *p* < 0.001), and the high-risk group predicted poor survival outcome, in line with the training dataset (Figures 5A,B). Similarly, both in the GSE2658 (Figure 5C) GSE136337 datasets (Figures 5D,E), the high-risk groups showed significantly shorter OS than the low-risk groups and the log-rank *p* values were <0.0001, 0.00017, and 0.012, respectively. Taken together, our five-gene risk score model was confirmed to be an independent prognostic factor in three testing datasets and can effectively predict the prognostic risk of MM patients.

### Identification of Genetic Risk Indicators

**FIGURE 6:**
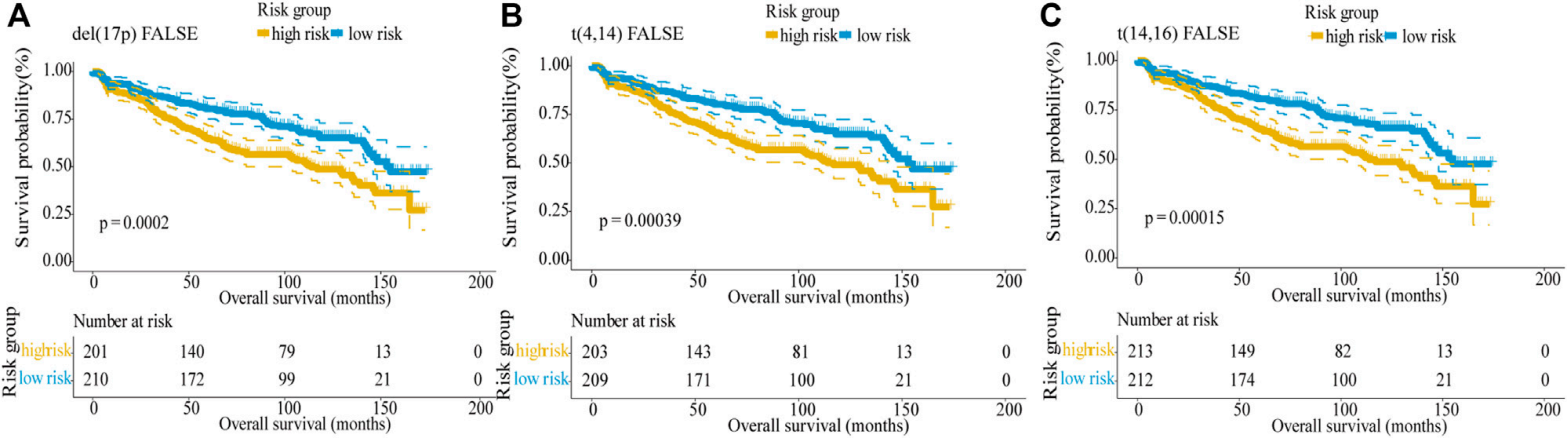
Validation of the five-gene risk score model in patients without genetic risk indicators by Kaplan–Meier curves. **(A)** MM patients without del (17p) (*p* = 0.0002). **(B)** MM patients without t (4,14) (*p* = 0.00039). **(C)** MM patients without t (14,16) (*p* = 0.00015). MM patients were divided into high-risk and low-risk groups by the median risk score.

Del (17p), t (4; 14), and t (14; 16) are defined as high-risk genetic factors by the IMWG. To verify the predictive ability of the five-gene risk score model in patients with or without these genetic factors, we analyzed the GSE136337 dataset which contains these indicators. First, we divided MM patients into two subgroups based on absence/presence del (17p), and the number of these patients were 411 and 15, respectively. Then, the patients were further subdivided into high-risk and low-risk groups by the median risk score. Kaplan–Meier curves of PFS showed no difference between the del (17p) FALSE and del (17p) TRUE group (log-rank *p* = 0.86), which indicated that del (17p) was not an effective indicator for prognostic outcome (Supplementary Figure S2A). However, in patients without del (17p), the difference between two groups was statistically significant (log-rank *p* < 0.0001, Figure 6A), with the high-risk group showing shorter overall survival than the low-risk group, whereas in patients with del (17p), the difference was not significant (log-rank *p* = 0.49; Supplementary Figure S2B).

Similarly, the results showed that there was no difference between the t (4,14) FALSE and t (4,14) TRUE group, indicating t (4,14) was not an effective indicator for prognostic outcomes (log-rank *p* = 0.98, Supplementary Figure S2C). In patients without t (4,14), the low-risk group showed higher overall survival than the high-risk group (log-rank *p* < 0.0001; Figure 6B). But for patients with t (4,14), the difference between the two groups was not significant (log-rank *p* = 0.1; Supplementary Figure S2D). Since there was only 1 MM patient with t (14,16), t (14,16) also cannot be used as a predictor of prognosis (log-rank *p* = 0.48, Supplementary Figure S2E). For 425 MM patients without t (14,16), the result showed that the survival was longer in the low-risk group than in the high-risk group (log-rank *p* < 0.0001; Figure 6C). In conclusion, our five-gene risk score model could effectively predict the prognosis of MM patients without high-risk genetic factors.

### Identification of Clinical Risk Indicators

**FIGURE 7:**
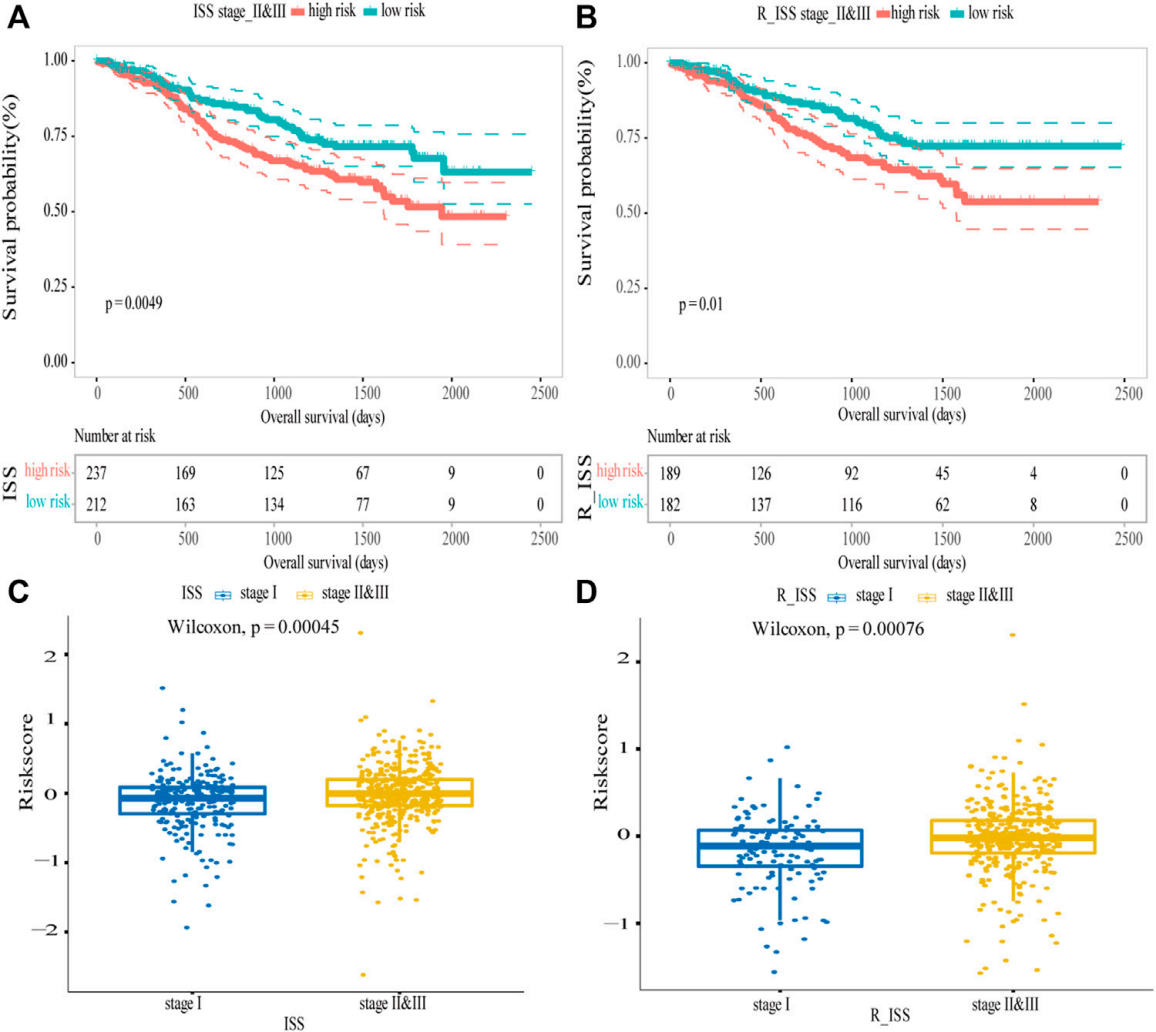
Validation of the five-gene risk score model in ISS and R_ISS. **(A)** Kaplan–Meier curves of MM patients in stage II and III of ISS (*p* = 0.0049). **(B)** Kaplan–Meier curves of MM patients in stage II and III of R-ISS (*p* = 0.01). **(C)** Survival differences between stage I and stage II and III of ISS (*p* = 0.00045). **(D)** Survival differences between stage I and stage II and III of R_ISS (*p* = 0.00076). MM patients were divided into high-risk and low-risk groups by the median risk score.

R_ISS and ISS were the widely used systems for the stratification of MM patients. In the MMRF dataset, the difference between different stages were highly significant (log-rank *p* < 0.0001; Supplementary Figures S3A,B). The ISS and R_ISS systems divided MM patients into three stages, and stage II and stage III were considered as progressive stages for MM patients. Thus, we verified the model’s predictive ability in the patients with these two stages, and the results showed that by combining the data from the ISS stage II and III, the difference in OS between the high-risk and low-risk groups was highly significant (log-rank *p* = 0.0049; Figure 7A), the survival of the low-risk group was longer than the survival of high-risk group. However, for the ISS stage I, the difference between the two groups was not discernible (log-rank *p* = 0.16; Supplementary Figure S3C). For R-ISS, we performed the same analysis, broadly consistent with the above. For stage II and III, the difference between the high-risk and low-risk groups was significant (log-rank *p* = 0.01; Figure 7B); stage I was not significant (log-rank *p* = 0.7; Supplementary Figure S3D). As the disease progressed, the risk scores became higher. The risk scores were significantly higher in stage II and III patients than in stage I patients in the ISS (Wilcoxon test *p* = 0.00045, Figure 7C) and R-ISS (Wilcoxon test *p* = 0.00076, Figure 7D). In addition, for ISS, when comparing stage II and stage III patients with stage I patients separately, the difference of risk scores between two stages were significant (Wilcoxon test p < 0.05). But the difference was not significant between stage II and stage III patients (Wilcoxon test p = 0.1). For R-ISS, when comparing stage I patients, stage II patients, and stage III patients separately, the risk scores between these stages were highly significant (Wilcoxon test p < 0.05, Supplementary Figure S3E,F). In conclusion, for high-risk ISS and R_ISS stage patients, the five-gene risk score model was confirmed to be an independent prognostic factor and can be further used to predict the prognostic outcome more accurately.

## Discussion

MM is a malignancy of terminally differentiated plasma cells, which in most cases remain incurable, the MM cells are mainly resident in the bone marrow (Kumar et al., 2017). Patients suffering from MM often display heterogeneous clinical outcomes, and MM remains a challenge due to the tendency to relapses for most patients (Sonneveld and Broijl, 2016). Among patients receiving the same treatments, survival outcomes can vary widely. In this regard, treatment options for individualized treatment are lacking.

Therefore, a prognostic signature beyond the current staging system is needed to establish to improve prognostic precision and guide clinical therapy. Many studies have found that gene transcription levels are closely related to tumor prognosis and biomarkers have been studied to improve prediction accuracy. In this study, we integrated public MM datasets and constructed a five-gene prognostic risk score model. The results demonstrated its validity in three independent datasets. The risk score model was confirmed to be an independent prognostic factor in multiple analyses that included genetic factors and clinical factors. Compared with other prognostic models, our model predicted survival outcomes effectively and were applied to predict the prognosis of patients with high-risk ISS/R-ISS stage or patients without high-risk genetic factors innovatively. As shown in Figure 6, for patients without del (17p), t (4,14), and t (14,16), the difference between two groups was statistically significant, the high-risk group showed shorter overall survival than the low-risk group.

The five genes in the risk score model: Endothelial PAS domain-containing protein 1 (*EPAS1*), often known as HIF2α, is a type of hypoxia-inducible factor (Tian et al., 1997). In colorectal carcinoma, the *EPAS1* protein expression inversely correlated with higher tumor grade and is associated with poor prognosis (Baba et al., 2010). *ERC2* (ELKS/RAB6-Interacting/CAST Family Member 2) is a protein-coding gene located in presynaptic active zones (Ko et al., 2006). In renal-cell carcinomas (Arai et al., 2014), frequent genetic and transcriptional inactivation of *ERC2* occurs, suggesting that *ERC2* may be involved in cancer progression. *PRC1* (protein regulator of cytokinesis-1) belongs to the microtubule-associated protein family and is involved in cytokinesis. *PRC1* was significantly overexpressed in breast cancer and lung adenocarcinoma (Shimo et al., 2007; Zhan et al., 2017), despite the possible molecular mechanisms have not been fully elucidated. *CSGALNACT1* (chondroitin sulfate N-acetylgalactosaminyltransferase 1) encodes a protein involved in glycos–aminoglycan chain synthesis and modifications. Studies showed that in myeloma, *CSGALNACT1* was under-expressed in MM cells compared to normal bone marrow plasma cells, which suggest that the overexpression of *CSGALNACT1* is associated with a good prognosis (Bret et al., 2009). *CCND1* (cyclin D1) is involved in regulating cell cycle and transcriptional processes. Previous studies have found that in myeloma, the dysregulation of *CCND1* is associated with oncogenic event in patients (Padhi et al., 2013), but further functional studies are needed to validate.

The advantage of our research is large sample sizes, five GEO datasets, and the MMRF dataset are used for system analysis; and a variety of algorithms are used to explore the optimal prognostic model. For survival information (OS, PFS, and EFS) in three testing datasets, the five-gene risk score model also acquired effective prognostic predictions; the survival of low-risk group was higher than the survival in the high-risk group. For genetic factors [del (17p), t (4; 14), and t (14; 16)] and clinical factors (ISS and R-ISS), adding the model can increase the prediction accuracy significantly. All these results demonstrate the stability and reliability of the model, which is an independent predictor of survival, can identify high-/low-risk patients and provide effective treatment recommendations. More importantly, in previous studies, these five genes have been confirmed to be associated with tumor, so further functional studies are needed to evaluate the roles of five genes in myeloma.

Despite the model is effective in predicting prognosis, there were still some shortcomings. First, we used multiple datasets, but there are biases between different platforms which may cause differences in results. Second, this is a retrospective study; further prospective studies are needed to confirm the results. Third, the contribution of each gene in the five-gene risk score model is unknown, further functional studies are needed to be validated.

## Data Availability

Publicly available datasets were analyzed in this study. These data can be found here: https://www.ncbi.nlm.nih.gov/geo/ https://research.themmrf.org/.
