# Supplementary material for: A Five-Gene Risk Score Model for Predicting the Prognosis of Multiple Myeloma Patients Based on Gene Expression Profiles
Source: Front Genet. 2021 Nov 30;12:785330. doi: 10.3389/fgene.2021.785330 (PMC8669596; doi:10.3389/fgene.2021.785330)
Supplement: Supplementary file 1 [file DataSheet1.ZIP › additional files/Supplementary Figure legend.docx]

**Supplementary Figure 1** Volcano plot of 304 differentially expressed genes and validation of four-gene model. (**A**) The volcano plot showed the DEGs between two groups with p < 0.05 and |log FC| > 1 as the threshold. The red dots represent 90 significantly upregulated genes, and the blue dots represent 214 significantly downregulated genes. (**B**) Validation of the four-gene model in GSE136337 datasets (P = 0.092).

**Supplementary Figure 2** Validation of the five-gene risk score model in patients with genetic risk indicators by Kaplan-Meier curves. (**A**) MM patients with/without del(17p) (P = 0.86). (**B**) MM patients with del(17p) (P = 0.49). (**C**) MM patients with/without t(4,14) (P = 0.98). (**D**) MM patients with t(4,14) (P = 0.1). (**E**) MM patients with/without t(14,16) (P = 0.48). MM patients were divided into high-risk and low-risk groups by median risk score. The difference between the two groups was tested by log-rank test.

**Supplementary Figure 3** Validation of the five-gene risk score model in ISS and R_ISS. (**A**) Kaplan-Meier curves of MM patients between three stages of R_ISS (P < 0.0001) in MMRF. (**B**) Kaplan-Meier curves of MM patients between three stages of ISS (P < 0.0001) in MMRF. (**C**) ISS stage I in MMRF (P = 0.16). (**D**) R_ISS stage I in MMRF (P = 0.7). (**E**) Survival differences between three stages of ISS. (**F**) Survival differences between three stages of R_ISS.

**Supplementary Table 1** 304 differentially expressed genes

**Supplementary Table 2** 38 potential genes based on LASSO regression model

**Supplementary Table 3** Univariate Cox regression analysis of the 20 genes.

**Supplementary Table 4** Multivariate Cox regression analysis of the 11 genes.
