# Supplementary figures and images for: A Five-Gene Risk Score Model for Predicting the Prognosis of Multiple Myeloma Patients Based on Gene Expression Profiles
Source: Front Genet. 2021 Nov 30;12:785330. doi: 10.3389/fgene.2021.785330 (PMC8669596; doi:10.3389/fgene.2021.785330)

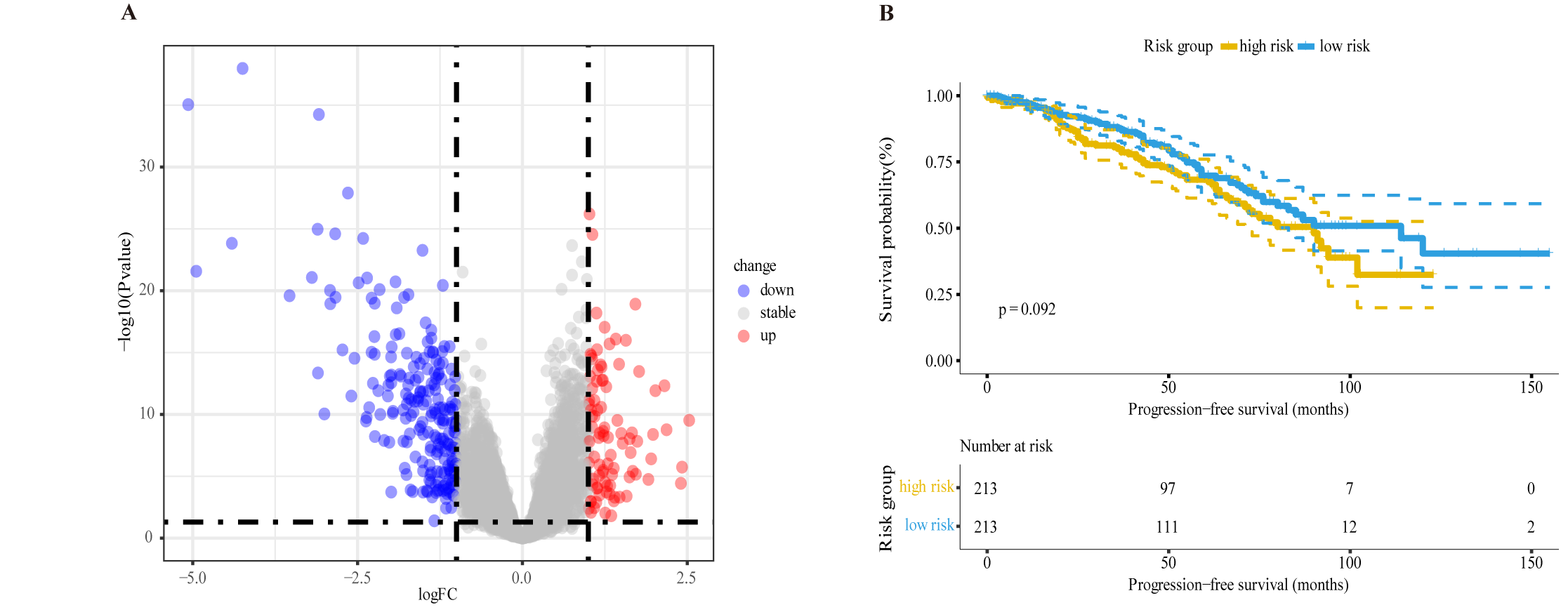

Supplement: Supplementary file 1 [file DataSheet1.ZIP › additional files/Supplementary Figure 1.tif]

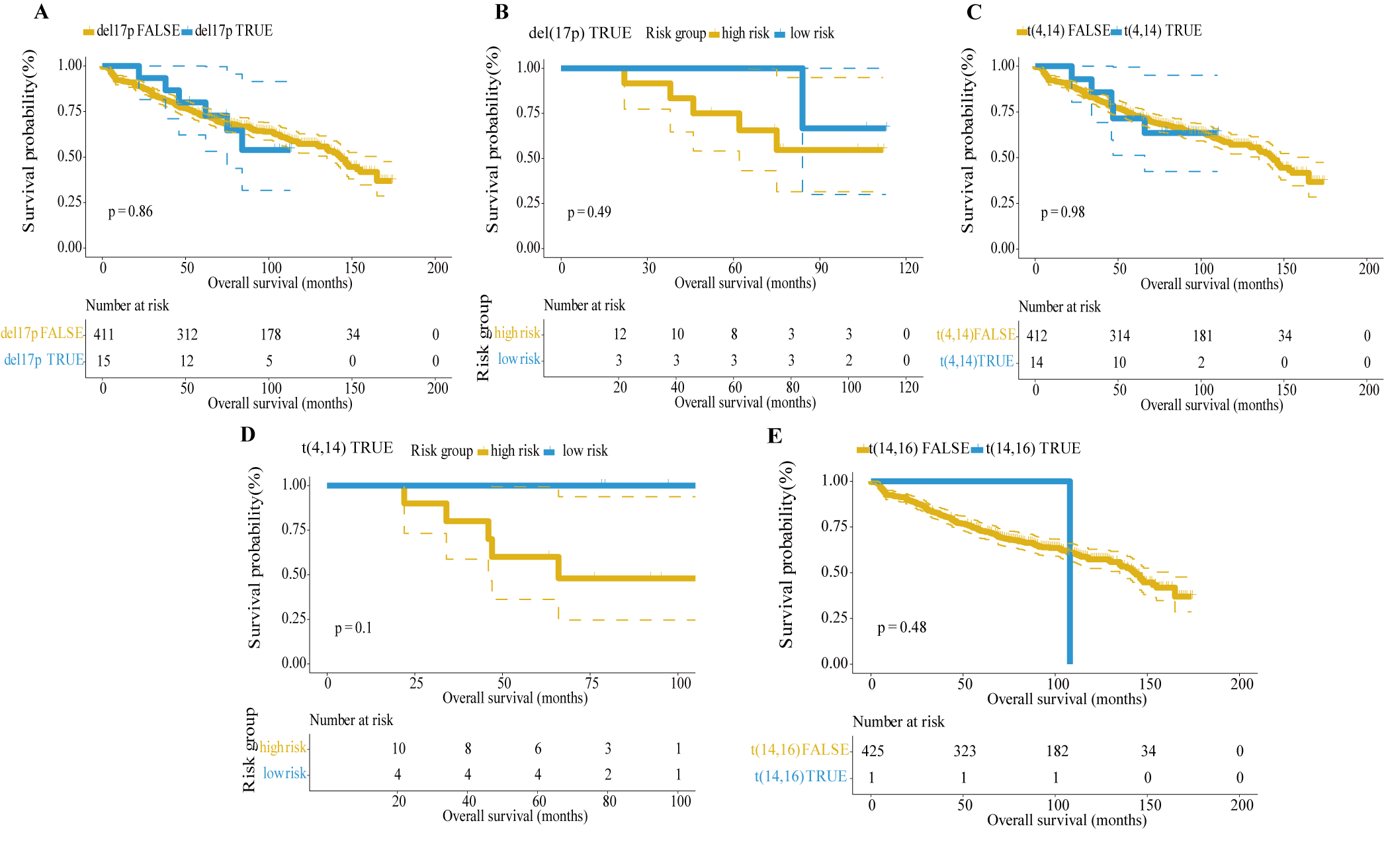

Supplement: Supplementary file 1 [file DataSheet1.ZIP › additional files/Supplementary Figure 2.tif]

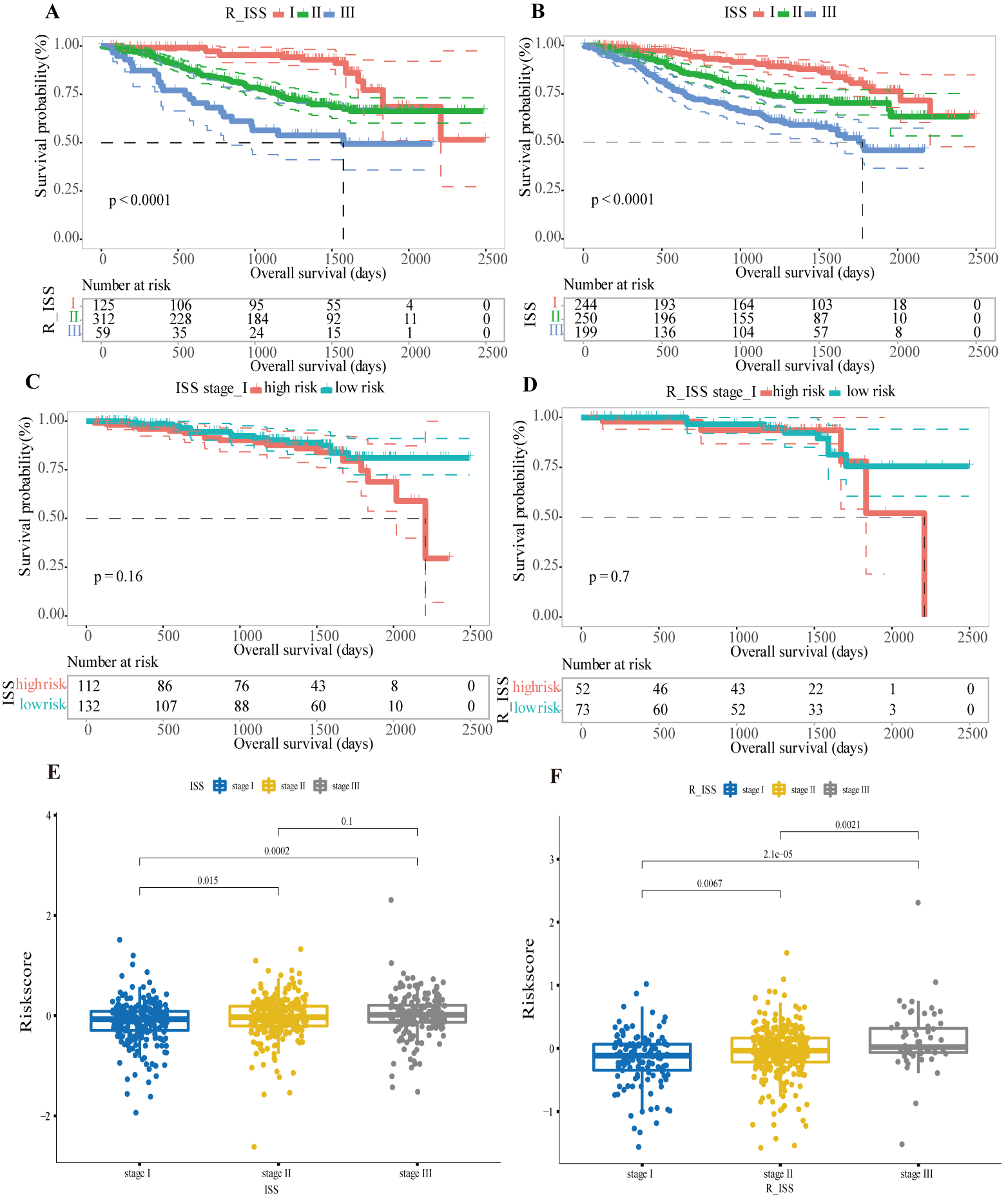

Supplement: Supplementary file 1 [file DataSheet1.ZIP › additional files/Supplementary Figure 3.tif]
